# Smart Graphite–Cement Composite for Roadway-Integrated Weigh-In-Motion Sensing

**DOI:** 10.3390/s20164518

**Published:** 2020-08-12

**Authors:** Hasan Borke Birgin, Antonella D’Alessandro, Simon Laflamme, Filippo Ubertini

**Affiliations:** 1 Department of Civil and Environmental Engineering, University of Perugia, via Goffredo Duranti 93, 06125 Perugia, Italy; hasanborke.birgin@unipg.it (H.B.B.); antonella.dalessandro@unipg.it (A.D.); filippo.ubertini@unipg.it (F.U.); 2 Department of Civil, Iowa State University, Construction and Environmental Engineering, Ames, IA 50011, USA

**Keywords:** smart materials, smart pavements, graphite, cement, weigh-in motion, strain

## Abstract

Smart multifunctional composites exhibit enhanced physical and mechanical properties and can provide structures with new capabilities. The authors have recently initiated a research program aimed at developing new strain-sensing pavement materials enabling roadway-integrated weigh-in motion (WIM) sensing. The goal is to achieve an accurate WIM for infrastructure monitoring at lower costs and with enhanced durability compared to off-the-shelf solutions. Previous work was devoted to formulating a signal processing algorithm for estimating the axle number and weights, along with the vehicle speed based on the outputs of a piezoresistive pavement material deployed within a bridge deck. This work proposes and characterizes a suitable low-cost and highly scalable cement-based composite with strain-sensing capabilities and sufficient sensitivity to meet WIM signal requirements. Graphite cement-based smart composites are presented, and their electromechanical properties are investigated in view of their application to WIM. These composites are engineered for scalability owing to the ease of dispersion of the graphite powder in the cement matrix, and can thus be used to build smart sections of road pavements. The research presented in this paper consists of electromechanical tests performed on samples of different amounts of graphite for the identification of the optimal mix in terms of signal sensitivity. An optimum inclusion level of 20% by weight of cement is obtained and selected for the fabrication of a plate of 30 × 15 × 5 cm^3^. Results from load identification tests conducted on the plate show that the proposed technology is capable of WIM.

## 1. Introduction

Cement, as the core component of concrete, is widely used in the construction of civil structures due to its easy production process, low cost, and suitable mechanical properties. Newly available additives and fillers for concrete can confer multifunctional properties to the material [1,2,3,4]. One possible functionality is strain-sensing, arising from enhanced piezoresistivity due to the presence of conductive fillers in nonconductive matrices [5,6]. This enhanced piezoresistivity occurs around the percolation threshold [7], defined as the filler concentration level at which the electrical phase transition takes place. Beyond that level, the conductive networks are formed inside the material matrix and the conductivity mode is governed by the networks of conductive fillers. It follows that the percolation threshold varies with the filler type and morphology. It can be identified through experimental [8,9], analytical [10,11], and numerical [12,13] approaches. Some popular conductive fillers used in the fabrication of self-sensing materials are carbon nanotubes [14,15,16,17,18], carbon black [17,19,20,21,22], graphene nanoplatelets (GNPs) [17,23], and carbon fibers [17,20,24,25]. Other less popular fillers have been researched, including nickel [26,27], aerographite [28], sodium silicate [29], and coal [30]. Hybrid doping methods were also reported by combining different types of fillers. Examples include carbon black and carbon fibers [31], carbon black and block-copolymer styrene-ethylene-butylene-styrene [21], carbon nanotubes and nickel fibers [32], carbon nanotubes and carbon nanofibers, carbon nanotubes and graphene, and carbon nanotubes and graphene nanoplatelets [33]. The fillers with higher aspect ratios were found to be more effective in terms of sensing capabilities and mechanical performance. Usually, they require a small amount of filler to reach percolation threshold. However, the fillers with higher aspect ratios tend to agglomerate and their dispersion over a large volume is difficult, thus generally limiting their applications to small scales [34].

Recent advances in nanotechnologies and smart materials have yielded numerous examples of traffic monitoring technologies for pavement-embedded self-sensitive sensors and sensor arrays [27,35,36]. The aim of this study is to improve on the field-applicability of self-sensing materials for weigh-in motion (WIM) sensing by developing a low-cost and highly scalable composite. The proposed technology leverages graphite powder as the filler. Graphite is an allotrope of carbon, known for its high electrical conductivity and low cost relative to other carbon-based conductive fillers [37]. The dispersion of graphite in the cement matrix can be satisfactorily achieved via mechanical mixing [38]. Highly conductive cement–graphite mixtures were studied for anode coatings [39], damage monitoring [40], and WIM applications [41]. Unlike rod-shaped carbon micro-/nanofibers and carbon nanotubes, graphite particles are oblate-shaped 3D fillers and possess lower aspect ratios [42]. They are considered as particle-shaped inclusions for which high amounts of doping are typically necessary to build conductive networks inside a material matrix [39].

Strain-sensing capabilities of composites with particle-shaped inclusions have been discussed in literature. For composite materials with GNPs, the layers of graphite [43] were found to have a percolating behavior analogous to that of rod-shaped fillers, resulting in increased sensitivity at a given filling level. However, the resulting gauge factor, or sensitivity of the electrical signal to strain, was found to vary with the magnitude of the load. The variation was explained by the imperfections inside the material matrix that are generated by the presence of GNPs [23]. Another particle-shaped filler, carbon black, was also found to increase the strain sensitivity of cementitious materials [21,44]. Unlike rod-shaped fillers, particle-shaped fillers like graphite, carbon black, and GNPs are known to decrease the mechanical properties of cementitious matrix [38]. Often, excessive concentration levels of these fillers are required in reaching the percolation threshold, resulting in unacceptable mechanical properties. Possible solutions are to add more water or to use plasticizers. Adding more water may also result in significant reductions in mechanical properties. The effect of adding water can cause particle agglomerations, but their effect on the piezoresistive property is uncertain, with conflicting results reported in [45] and [46]. The use of dispersants to minimize agglomerations may produce a decrease of mechanical properties [47] and sensitivity by modifying electrical characteristics [48]. Lastly, the use of plasticizers may yield noisy signals [47,49].

In a first study on self-sensing smart materials, the authors have numerically studied an algorithm enabling WIM for estimating the axle number and weights along with the vehicle speed [50]. Here, research is extended to studying the electromechanical properties of scalable, graphite-based smart materials to empower the formulation of more accurate models and field deployments of the WIM technology. Analytical and experimental investigations of the percolation threshold as a function of graphite concentration levels are conducted on small-scale cubic samples. These samples are tested under different levels of compressive loads to observe the variation in sensitivity and linearity with respect to various graphite content, and to identify an optimum graphite concentration level. A plate sample is then produced with the optimum level and subjected to load-controlled experiments, mimicking the effect of a wheel to investigate the material’s capability to identify and quantify the applied loads.

The rest of the paper is organized as follows. Section 2 describes the fabrication procedure and the testing equipment. Section 3 studies the identification of the percolation threshold. Section 4 discusses the electromechanical tests and the identification of the optimum doping level. Section 5 presents and discusses the mechanical performance of the composite. Section 6 presents the production process for the plate sample and discusses the experimental results. Section 7 concludes the paper.

## 2. Materials and Methods

This section presents the methodology in investigating the performance of graphite–cement composites in view of their applications to WIM. First, small-scale cubic samples are fabricated with different concentration levels of inclusions to study the percolation threshold and identify the optimum mixture for sensing purposes. Second, a plate sample of larger dimensions is fabricated using the identified optimal mixture and tested to study its load estimation capabilities.

### 2.1. Materials

**Figure 1:**
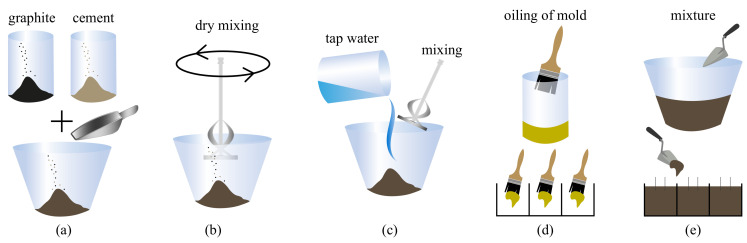
The production steps of the cement–graphite composite samples: (**a**) adding graphite and cement; (**b**) mixing the compounds in their dry powder form; (**c**) adding water and mixing until homogeneity; (**d**) preparing molds; (**e**) casting compound into the molds and electrodes.

The components of the smart composite material are Portland Cement (42.5 R), tap water, and graphite powder. The fabrication process of samples is as follows. Cement and graphite powders in corresponding proportions are mechanically mixed in their dry form, shown in Figure 1a,b. After homogeneity is attained, water is added slowly and mixing continues until the compound exhibits no agglomeration of powders, as shown in Figure 1c. Metal molds of 5 × 5 × 5 cm${}^{3}$ size are prepared and oiled to ensure easy demolding. Each mixture is cast into three molds, and two stainless steel nets are placed into each sample to form the electrodes, as shown in Figure 1d,e.

Table 1 reports the weights of material components used in fabricating the samples. The selected mixes differ in the graphite-to-cement weight percentage, from 0 to 40% with 10% increments. The water-to-cement weight ratio is initially 50% for the 0, 10, and 20% graphite-to-cement ratio mixtures, and 55% for the 30 and 40% graphite-to-cement ratio mixtures to increase the workability. Samples with 40% graphite-to-cement have been observed to lose their binding characteristics and are thus considered as having reached the limit of filler concentration.

### 2.2. Samples Characteristics

**Figure 2:**
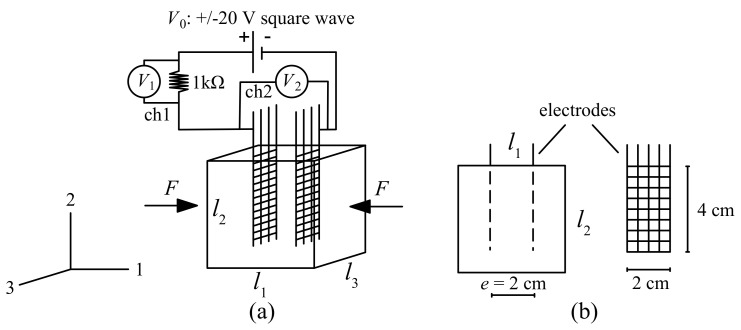
(**a**) Illustration of a cubic sample, loading, and data acquisition; (**b**) steel net electrodes and their placement.

**Figure 3:**
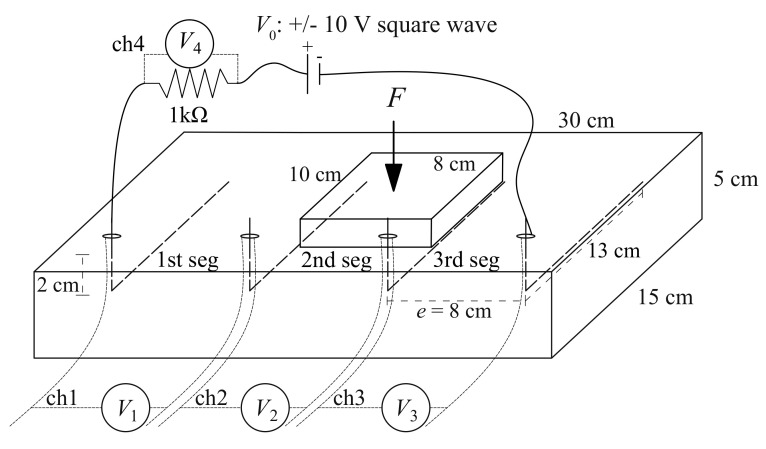
Illustration of the plate sample including the electric circuit, placement of the load *F*, and voltage acquisition channels (ch1 to ch4).

The cubic samples for this study are illustrated in Figure 2a. They have dimensions $l_{1}=l_{2}=l_{3}=5$ cm. Two steel net electrodes are placed at a mutual distance e=2 cm, Figure 2b, to connect the sample to the electrical circuit. The testing compression load *F* is applied onto both sides parallel to the electrodes. Taking a shunt resistor of 1 kΩ, the resistance of the sample cube, *R*, in Ohms, is written:

(1) $$R=1000\frac{V_{2}}{V_{1}},$$

where $V_{1}$ is the voltage reading taken over the shunt resistor obtained through channel 1 (ch1) of the experimental setup, and $V_{2}$ is the voltage reading taken over the sample obtained through channel 2 (ch2) of the experimental setup. The plate sample is illustrated in Figure 3. The electrodes are copper wires placed at a mutual distance of 8 cm, embedded 13 cm into the plate at a depth of 2 cm from the top surface. The choice of copper wires produce a particularly suitable result for the plate sample and for real-scale applications. The plate sample has a cross section of 30 × 15 cm${}^{2}$ and a thickness of 5 cm. The voltage readings are taken from each segment of the plate using a 1-kΩ shunt resistor connected in series. Equation (1) is used to calculate the segment resistance. The compression force *F* is applied onto the second segment of the plate.

For the voltage input, biphasic square waves were selected (see Figure 2 and Figure 3) to reduce signal drifts caused by the polarization of dielectric cement matrix [51].

### 2.3. Experimental Methodology

The electromechanical tests are carried out by mechanically loading and unloading the test samples that are electrically charged by a power source. Loads, deformations, and changes in electrical voltage are recorded. The variations in electrical resistance obtained from the tests using Equation (1) are then compared to the strain variations.

**Figure 4:**
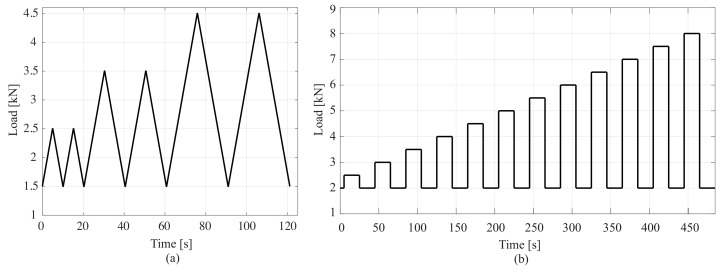
Loading histories for the electromechanical tests. (**a**) Cyclical load history used for cubic samples; and (**b**) step load history used for the plate sample.

Two different load patterns were adopted for loading the samples. For the cubic samples, in addition to the compressive preload of 1.5 kN, triangular cyclic compressive loads along direction-1 (Figure 2a) were applied to the samples with 1-, 2-, and 3-kN incremental peaks corresponding to incremental pressures of 0.4-, 0.8-, and 1.2-MPa, respectively. The pattern is plotted in Figure 4a.

For the smart composite plate, the adopted load pattern, plotted in Figure 4b, consists of a box load of increasing magnitude from a preload of 2 kN, with magnitudes increasing from 2.5 to 8 kN in 0.5 kN increments, corresponding to pressures ranging from 0.3125 MPa to 1 MPa in 0.0625 MPa increments. These pressure levels are in accordance with the fatigue traffic loads defined by Eurocodes [52].

The data acquisition system consists of a chassis, model NI PXIe-1092, equipped with a source measuring unit NI PXIe-4138 and 32-channel Analog Input Module NI PXIe-4302 controlled by a NI PXIe-8840 unit. Voltage readings were taken by different channels (ch) as indicated in Figure 2a and Figure 3. The data acquisition frequency of the potential difference is 10 Hz. Voltage square waves of 10-V and 20-V amplitudes and 1-Hz frequency were applied by a NI PXIe-4138. The load *F* was applied on a rectangular surface 8 × 10 cm${}^{2}$ using an electric servo testing machine, model Advantest 50-C7600 by Controls, equipped with a servo-hydraulic control unit, model 50-C9842, with a maximum load capacity of 15 kN. Strains were measured with three LVDT transducers with 10-mm maximum travel distance placed at 120 degrees in-plane. To this end, a preliminary calibration was conducted against 20 mm-long electric strain gauges attached on two opposite faces of four benchmark specimens in order to compensate the effect of the electrodes and LVDT imprecisions over very small displacements. All the tests were conducted in a laboratory under constant temperature conditions.

## 3. Evaluation of Electrical Properties of the Composites: Percolation Threshold

In this section, the percolation threshold is studied as a function of the amount of filler. The investigation is conducted both analytically and experimentally.

### 3.1. Analytical Investigation

The analytic derivation of the percolation threshold is adopted from reference studies [6,10,11]. In principle, the volume occupation of the fillers increases the number of contacts between conductive particles. Electrical percolation is attained when the average number of contacts per conductive particle is higher than a given threshold, estimated in literature at 1.38 [11]. The volumetric fraction of the conductive particles that yield such threshold is termed the critical volumetric fraction of percolation. Above this fraction, additional conductive particles will not substantially contribute to the conductivity. Below this fraction, the conductivity is expected to be significantly low. The main variable affecting percolation is the aspect ratio of the particles, defined as the ratio of the longer to the shorter dimension of the particles.

**Figure 5:**
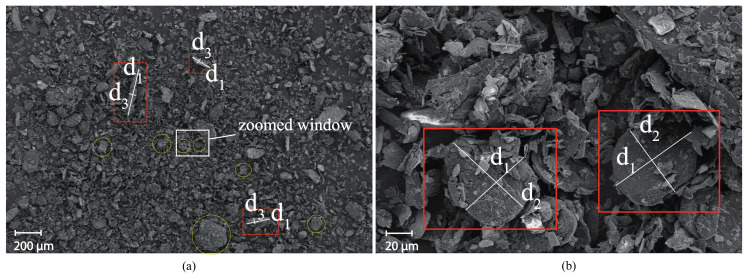
(**a**) SEM micrograph of graphite powder with (**b**) zoomed window. Labeled dimensions d${}_{1}$, d${}_{2}$, and d${}_{3}$ are the major axis length, minor axis length, and height of the elliptical cylinders, respectively.

Here, to analytically compute the volumetric fraction, the morphology of the graphite filler is inspected under scanning electron microscope (SEM), shown in Figure 5. From the SEM micrograph, the majority of the particles appear in the form of thin elliptic cylinders. The aspect ratio, AR, is taken as

(2) $$AR=\frac{d_{1}+d_{2}}{2d_{3}},$$

where d${}_{1}$ and d${}_{2}$ are major and minor axes of the elliptic surface, respectively; and d${}_{3}$ is the third axis dimension, the height of the elliptical cylinder. The resulting average AR is approximately 8 to 10. Therefore, the percolation is expected referring to work in [12], where critical volume fractions of fillers were calculated by 3D numerical simulations for a variety of aspect ratios; based on that work, the expected percolation threshold is to occur between 10% and 20% graphite-to-cement weight ratio.

### 3.2. Experimental Investigation

**Figure 6:**
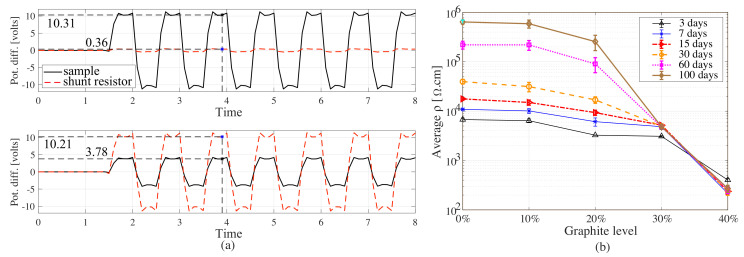
(**a**) Time series of potential difference (pot. diff.) from shunt resistor and the 20% (top) and 30% (bottom) graphite samples along with voltage values read from the biphasic time history; and (**b**) resistivity versus curing time for samples with 0, 10, 20, 30, and 40% of graphite inclusions.

The resistivity values of samples have been evaluated by connecting the samples to the readout circuit without the application of any external load, and recording voltage differences. The variations of the voltage drop across the shunt and the 20 and 30% graphite samples after 100 days of curing are reported in Figure 6a. The resistivity, ρ, of the material is calculated using

(3) $$\rho =R\frac{A}{e},$$

where *A* is the cross-sectional area of the sample between the electrodes, *e* is the distance between electrodes, and *R* is the resistance of the sample.

Figure 6b plots the average resistivity and range from three samples under each graphite concentration level at 3, 7, 15, 30, 60, and 100 days of curing time. The increase in resistivity during curing is the result of drying and chemical transformations resulting in reduced ionic conduction. Samples with 40% graphite-to-cement exhibit a lower resistivity than the other samples during curing. From the figure, it can be observed that the electrical behavior transition starts at approximately 20% graphite-to-cement loadings, after which the resistivity starts to drops rapidly, in particular beyond 60 days of curing. This value is in good agreement with analytical predictions presented in the previous subsection and with the values reported in the literature [53,54].

## 4. Sensing Investigation of Graphite–Cement Composites

In this section, the effects of graphite inclusions on electrical conduction and strain sensitivity of material are investigated through electromechanical tests. First, the electromechanical model is derived, followed by a presentation and discussion of results.

The electromechanical model of strain-sensing smart composite can be derived as follows. Referring to Figure 2, Equation (3) can be written:

(4) $$R=\rho \frac{e}{l_{2}\cdot l_{3}}.$$

Under uniaxial strain, the change in the resistance can be formulated under the assumption of isotropic material:

(5) $$\begin{array}{cc} \frac{\Delta R}{R} & =\frac{\Delta \rho }{\rho }+\frac{\Delta e}{e}-\frac{\Delta l_{2}}{l_{2}}-\frac{\Delta l_{3}}{l_{3}} \end{array}$$

(6) $$\begin{array}{cc} \; & =\frac{\Delta \rho }{\rho }+\left(1+2\nu \right)\varepsilon_{1}, \end{array}$$

where ν is the Poisson’s ratio of the composite and ε denotes strain along the associated axis in subscript, *R* is the electrical resistance obtained during tests, and Δ denotes a variation. The gauge factor, λ, is defined as the ratio of relative change in resistance to strain, written as

(7) $$\lambda =-\frac{\frac{\Delta R}{R}}{\varepsilon_{1}}=-\frac{\frac{\Delta \rho }{\rho }}{\varepsilon_{1}}-\left(1+2\nu \right),$$

where $\varepsilon_{1}$ is taken positive in compression, with compression provoking a decrease in relative resistance.

**Figure 7:**
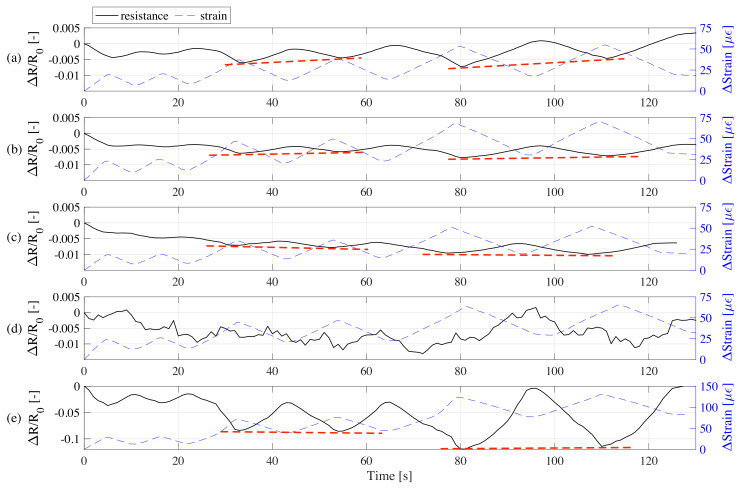
Typical time series of relative change in resistance and strain loading under each sample: (**a**) 0%; (**b**) 10%; (**c**) 20%; (**d**) 30%; and (**e**) 40% graphite-to-cement ratios.

Figure 7 plots the time history of strain and the normalized electrical resistance obtained during electromechanical tests on samples with different graphite-to-cement ratios. As expected from the electromechanical model, the relative resistance and strain exhibit opposite trends. Despite the adopted biphasic measurement method, the residual drift due to polarization is evident under the 0% graphite-to-cement samples, because of the governing dielectric behavior. The dashed red line in the figure shows the drift between two adjacent minima, highlighting significant drifts for 0 and 10% graphite-to-cement ratio samples. Samples with a higher amount of graphite do not show significant drifts unrelated from strain time history. The recorded signal from the 30% samples exhibit major distortions. That is attributable to the exceedance of the percolation threshold, where compressive loads cause minimum changes in conductivity. Nevertheless, the 40% samples manifest a good sensitivity but high nonlinearity. This can be attributed to the material imperfections (voids and agglomerations) due to overloading of conductive fillers. Moreover, residual deformations in the strain time history are evident for all the samples with 40% of graphite. The electrical outputs of samples with 10 and 20% graphite-to-cement appear consistent with the strain time history.

**Figure 8:**
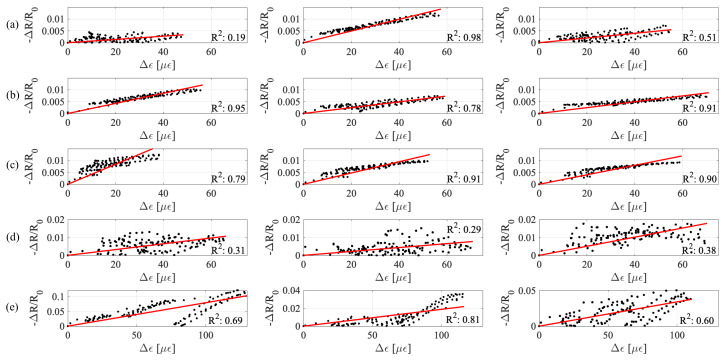
Relative change in resistance versus strain for all samples, along with best linear fit lines: (**a**) 0%; (**b**) 10%; (**c**) 20%; (**d**) 30%; and (**e**) 40% graphite-to-cement ratios.

Figure 8 shows the linearity of the signals obtained from electromechanical tests for every sample. Samples at 10 and 20% graphite-to-cement exhibit higher linearity, whereby 20% samples show higher sensitivity observed through a steeper linear fit. Table 2 summarizes results from the electromechanical tests. Consistent with results of Figure 7, the 40% samples yield higher sensitivity than the 30% samples, yet very high fitting error.

Values of sensitivity, or gauge factors, are consistent with literature [50] and increase with increasing graphite-to-cement ratio up to 20%, reaching a maximum at 20% if results from the 40% are ignored due to the unreliability of the samples. This is in agreement with the analytical results. The 20% ratio is selected as the optimal mix.

## 5. Mechanical Properties of Graphite–Cement Composites

**Figure 9:**
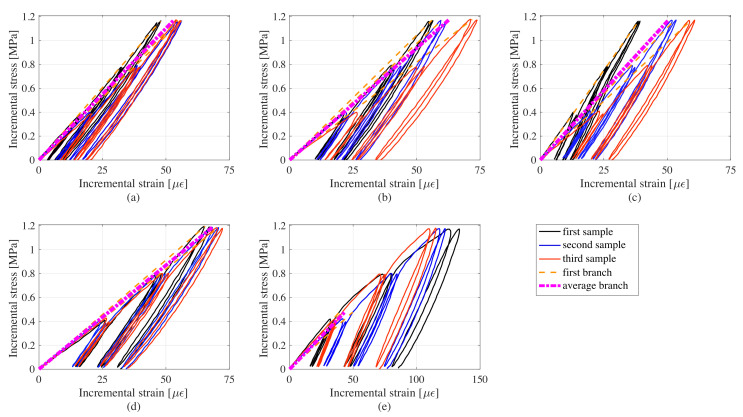
Stress versus strain curves for all samples: (**a**) 0%; (**b**) 10%; (**c**) 20%; (**d**) 30%; and (**e**) 40% graphite-to-cement ratios.

Mechanical properties of samples under different graphite-to-cement ratios were evaluated using the cyclic load tests described in the previous sections. Figure 9 plots incremental stress versus incremental strain data for every samples. Stress is calculated by dividing the force, *F* to the cross-sectional area of 25 cm${}^{2}$. The dashed black lines represent the loading branch taken to estimate the Young’s modulus under the assumption of being in the elastic range of the material. The magenta dot-dashed line is the mean stress-strain branch.

From Figure 9, it can be observed that the stress-strain curves for the 40% samples exhibit plastic deformations around the stress increment of 0.5 MPa, confirming the hypothesis that these samples were overloaded with filler and had unacceptable electromechanical performance. Another observation is that the 0% samples have similar loading and unloading moduli, whereas those of the other samples have considerably higher unloading moduli compared to the loading moduli.

Table 3 lists the minimum and average elastic moduli E obtained under each graphite-to-cement ratio. Results reveal a significant change in the elastic modulus under the 40% samples. For the other samples, the elastic moduli are in the same order, with a slight increase for the 20% samples, and variations in results may be attributed to the hand-fabrication process. The minimum values for elastic moduli are more than those for various pavement materials (3.5–13.5 GPa) reported in [55].

Overall, results demonstrate that the 20% graphite-to-cement composite provides adequate mechanical performance and is suitable as a structural pavement material. This optimal mix was used to fabricate the smart composite plate, discussed in what follows.

## 6. Self-Monitoring Investigation of a Cementitious Plate Element with Graphite

A smart plate sample was fabricated to be more representative of a self-sensing pavement unit for WIM applications. The load-sensing capability of the composite plate can be achieved by tracking the electrical resistance of segments between electrodes, as illustrated in Figure 3. The utilized testing equipment is the same as that used for testing the cubic samples. Three high precision LVDTs were added around the plate to record the displacement of the top of the plate during tests.

The materials listed in Table 4 were mixed to produce a plate sample of dimensions 30 × 15 × 5 cm${}^{3}$ with 20% graphite-to-cement weight ratio. The electrode configuration, loading, and data acquisition are shown in Figure 3. Loads were distributed over the corresponding area using a steel plate of dimensions 10 × 8 × 2 cm${}^{3}$ to simulate the scaled wheel contact area. The sample was electrically charged from the external electrodes. The central segment was selected to remove the effects of contact resistance in estimating loads [56].

### 6.1. Investigation of the Polarization Behavior

**Figure 10:**
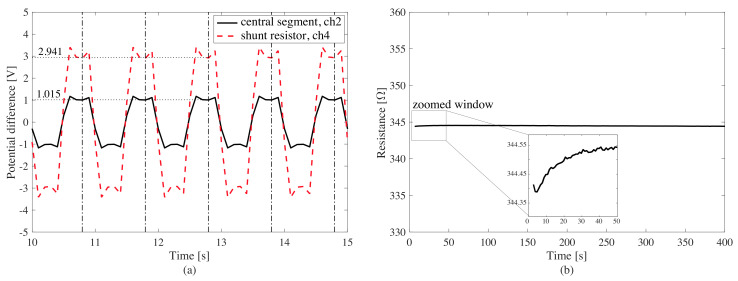
Plate response to the unloaded polarization test: (**a**) time window of raw voltage reading from ch4 (shunt resistor) and ch2 (central segment); (**b**) resistance time history of the central segment of the plate with a zoom on the first 50 s of the signal.

Two tests were conducted to evaluate the importance of the polarization effects, both under different loading conditions. During the first test, the plate was kept unloaded and the potential difference was applied. Results are shown in Figure 10. The biphasic voltage signal coming from the shunt resistor and the central segment does not exhibit noise nor temporal shifts. The 80% charge points of square wave were selected and indicated in Figure 10a, and the resistance of the central segment was calculated via proportional comparison with the read voltage from shunt resistor. The data shows a stable resistance time history in Figure 10b. As displayed in the zoomed window, in the first seconds of charging, the observed resistance of the central segment increases until a steady-state is achieved due to the capacitance behavior of the material.

**Figure 11:**
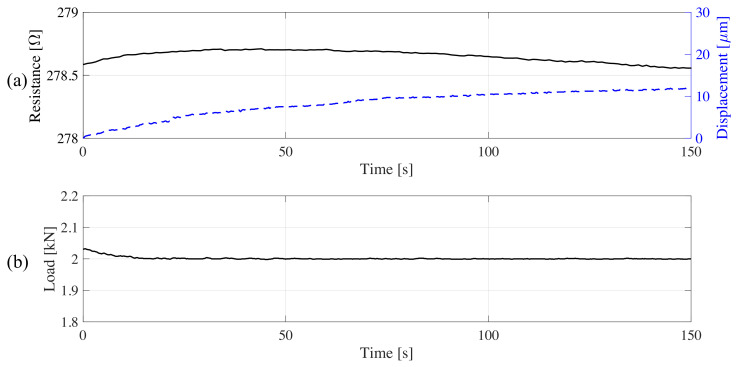
Plate response to the loaded polarization test: (**a**) resistance and displacement of the top of the plate; (**b**) load time history.

During the second test, the plate was loaded under 2 kN for 150 s. Using the same postprocessing, the resistance time history was obtained. Results are shown in Figure 11. A comparison of the resistance and displacement time histories reveal a nonlinear drift, attributable to the viscoelastic nature of the composite material responding to a long duration load. Given the envisioned WIM application, the drift due to residual strain caused by traffic load is expected to be negligible.

### 6.2. Evaluation of the Plate Sensitivity to Load Tracking

**Figure 12:**
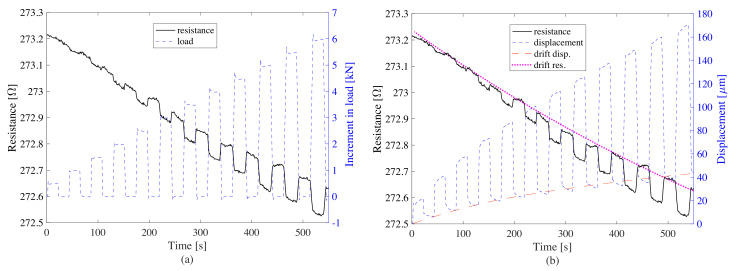
Raw time histories of electromechanical test with increasing box loads: (**a**) resistance versus increment in load; (**b**) resistance versus displacement of the top of the plate.

Figure 12a,b show the resistance time history against load magnitude and top displacement, respectively. Data was postprocessed by removing the nonlinear drift in the resistance (magenta dashed line) and displacement (red dashed line) time series, and normalizing the resistance data to its first value. The nonlinear drift was identified by fitting and subtracting a polynomial of second degree from the data.

**Figure 13:**
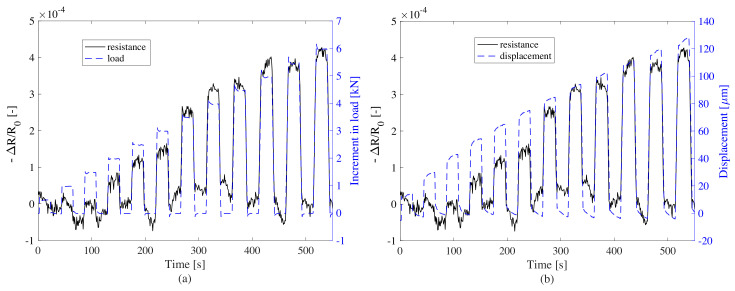
Postprocessed time histories of electromechanical test with increasing box loads: (**a**) change of resistance versus change of load; (**b**) relative resistance versus displacement of the top of the plate.

**Figure 14:**
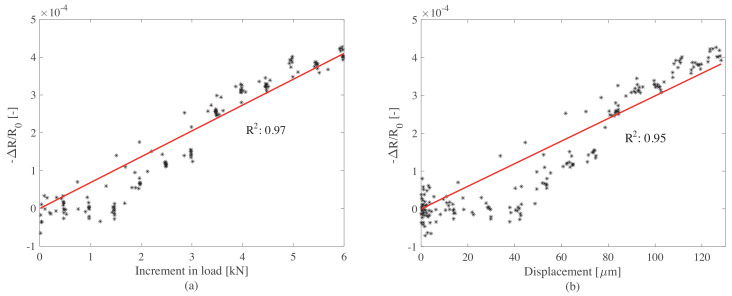
Linearity of the electrical signal with respect to (**a**) change in load and (**b**) change in displacement of the top of the plate.

The postprocessed data is plotted in Figure 13a,b. Figure 14 shows the linearity of the signal as a function of changes in loads (Figure 14a) and displacement (Figure 14b). Results show a good linearity with respect to both load variations and displacements. The noise-to-signal ratio is observed to be low after the load increment of 2 kN, which in terms of pressure is 0.25 MPa. When compared to the traffic loads, this threshold is lower than the average truck axle pressures defined in [52]. Hence, the precision of the WIM system is expected to be high, especially when measuring the weight of trucks. The response of the plate to the load increment is observed to be rapid and therefore convenient for rapid traffic loads. These results indicate that the smart composite plate could be used to estimate the change in pressure caused by an external load by inspecting the change in electrical resistance.

### 6.3. Discussion

Tests conducted in this work yielded the following observations on the physical, mechanical, and electrical properties of cement-based composites doped with graphite powder: (1) the dispersion of the graphite in a water-based cementitious matrix using a simple mechanical mixing process appeared effective, resulting in a scalable fabrication process to produce large components and for in-situ applications; (2) tests on the cubic samples with different percentages of graphite permitted the identification of the most performing mix, where it was found that a 20% graphite-to-cement weight ratio yielded a better linearity ($R^{2}$ = 0.87) and higher gauge factor compared with other acceptable mixes; (3) the study on a smart plate fabricated using the identified optimal mix showed a good fit between the change in resistance and load increment ($R^{2}$ = 0.97), and between the change in resistance and displacement of the plate ($R^{2}$ = 0.95), demonstrating promise for field applications; (4) the acquired signals on the smart plate exhibited low noise-to-signal ratio, therefore showing promise for WIM sensing; and (5) the investigated composite exhibited good mechanical properties, with no significant reduction in the Young’s modulus encountered during the electromechanical tests.

## 7. Conclusions

This study proposed the use of graphite inclusions within cement matrix for low-cost and easily scalable roadway integrated WIM applications. Graphite inclusions are scalable, easy to disperse, and low-cost compared to other conductive micro- or nanofillers used in creating smart composites such as carbon nanotubes and nanofibers. The conclusions of the study are as follows: (i) 20% graphite-to-cement weight ratio (12% volumetric ratio) is the optimum doping level in terms of strain sensitivity for cementitious materials; (ii) the elastic properties of the proposed material are comparable with those of other more traditional pavement materials; (iii) sensitivity tests conducted using a medium-scaled sample demonstrated that the proposed material can be used to estimate an applied load. Overall, results of the present study demonstrated that the proposed composite material and measurement method could be readily applied in the field for weigh-in motion sensing.

## Figures and Tables

**Table 1:** Material components for graphite–cement samples.

Component	Resistivity (Ω· cm)	Density (g/cm${}^{3}$)	Component Weight (g)				
Cement-Dry	–	1.5	636	636	636	636	636
Tap water	2000	1.0	318	318	318	350	350
Graphite	3–5$\times 10^{-4}$	1.2	0	64	127	191	254
Weight fraction of graphite to cement (graphite-to-cement)			0%	10%	20%	30%	40%
Volume fraction of graphite			0%	6.7%	12.2%	17.3%	21.8%

**Table 2:** Experimental results from the electromechanical tests, where λ is the gauge factor, $R^{2}$ is the coefficient of determination, and 95% fit interval is the interval that contains 95% of best fitting data points.

Graphite %	0%			10%			20%			30%			40%		
Sample #	1	2	3	1	2	3	1	2	3	1	2	3	1	2	3
λ	31	247	88	213	123	124	422	237	200	160	72	254	794	170	296
Average λ	122			153			286			162			420		
95% fit interval	27	13	23	12	11	9	44	17	13	31	39	38	110	44	83
Average 95% int.	21			11			25			36			79		
Linearity, $R^{2}$ (%)	19	98	51	95	78	91	79	91	90	31	29	38	69	81	60
Average Linearity	56			88			87			33			70		

**Table 3:** Young’s modulus (E) of samples.

Graphite	0%		10%		20%		30%		40%	
	min	avg	min	avg	min	avg	min	avg	min	avg
E (GPa)	21	22	16	19	20	24	16	17	9	11

**Table 4:** Materials used in fabricating the smart plate.

Component	Quantity (g)
Cement	3500
Graphite	700
Tap Water	1750
